# Focal Intramucosal Adenocarcinoma Occurring in Gastric Hyperplastic Polyps Treated with Endoscopic Mucosal Resection

**DOI:** 10.1155/2018/7431290

**Published:** 2018-09-24

**Authors:** Jéssica Alférez-Andía, Harold Benites-Goñi, Fernando Palacios-Salas

**Affiliations:** ^1^Hospital Santa Rosa, Lima, Peru; ^2^Hospital Nacional Edgardo Rebagliati Martins, Lima, Peru

## Abstract

Hyperplastic polyps are the most frequent benign epithelial gastric polyps. Although they are considered nonneoplastic, some cases have been reported with focal adenocarcinoma. We present the case of a 59-year-old woman with a sessile lesion of 15 mm on the distal gastric body associated with an extensive atrophic gastritis. Magnifying endoscopy with Fuji Intelligent Color Enhancement (FICE) revealed an irregular microsurface pattern at the apex, suggesting malignancy. A mucosectomy was performed. The histopathology revealed that the base corresponded to a hyperplastic polyp, where a tubular adenoma with high-grade dysplasia was established, with focal well-differentiated intramucosal tubular adenocarcinoma.

## 1. Introduction

Hyperplastic polyps (HP) are the most common nonneoplastic polyps in the stomach [1]. Although the pathogenesis of HP is not yet defined, its etiology seems to be associated with an inflammatory response [2]. HP are usually benign, so they are managed as such; however, there are some studies that describe the presence of dysplasia and even adenocarcinoma [3].

A case of gastric HP with focal adenocarcinoma is presented.

## 2. Case Report

A 59-year-old woman with epigastralgia was evaluated in gastroenterology and an upper digestive endoscopy was indicated. During the endoscopic examination a gastric polyp was found and the patient was referred to our hospital for treatment.

**Figure 1:**
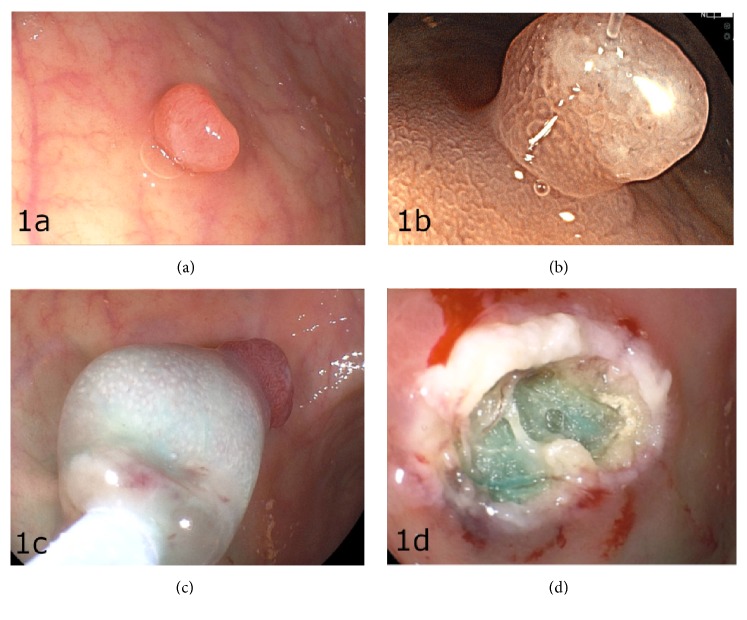
Endoscopic findings. (a) Endoscopic appearance of the sessile gastric HP. (b) Magnifying endoscopy with FICE of the polyp. (c) EMR with lateral safety margins. (d) Endoscopic finding after* en bloc* resection.

Endoscopic examination of the upper digestive tract revealed extensive atrophic gastritis and a sessile lesion of 15 mm of reddish coloration in the distal gastric body (Figure 1(a)). Magnifying endoscopy with Fuji Intelligent Color Enhancement (FICE) of the polyp showed an irregular microsurface pattern at the apex, noticing a demarcating line, highly suggestive changes of malignancy (Figure 1(b)). Endoscopic mucosal resection (EMR) was performed with lateral safety margins (Figures 1(c) and 1(d)).

**Figure 2:**
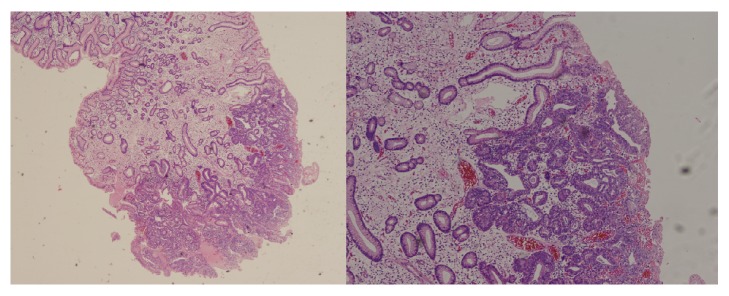
Pathologic findings. (A) Adenomatous change in the background of hyperplastic polyp (H&E, ×12.5). (B) Foci of carcinomatous transformation in the adenomatous lesion (H&E,×40).

The histopathological evaluation of the resected polyp revealed that the base corresponded to a hyperplastic polyp, in which a tubular adenoma with high-grade dysplasia was established with focal well-differentiated intramucosal tubular adenocarcinoma (Figure 2). The lesion was resected completely with a lateral margin greater than 2 mm. No evidence of lymphovascular invasion was noticed. For this reason, it was concluded that the EMR was successfully performed fulfilling the criteria of histological cure [4]. The polyp with focal adenocarcinoma was classified as early gastric cancer, type 0-Is according to the Paris classification, and T1a according to the TNM classification [5]. Histopathological evaluation of the surrounding mucosa revealed atrophic gastritis in the body and antrum, with no evidence of* Helicobacter pylori* infection.

## 3. Discussion

HP are inflammatory proliferation of gastric foveolar cells, which produce mucin and cover the gastric surface [6]. HP are the most common type of polypoid lesion in the stomach [1]. It usually occurs in people over 60 years of age with a slight female preponderance. Most patients are asymptomatic and are usually found incidentally [2]. However, some patients may present epigastralgia or upper gastrointestinal bleeding leading to anemia [7].

The etiology of these polyps is still not well defined, but it has been suggested that there may be an inflammatory process (for example, due to autoimmune gastritis or* Helicobacter pylori* infection), which develops on a reparative phenomenon in the form of foveolar hyperplasia; this hyperplastic tissue may disappear (24%) or persist, progressing to the formation of a hyperplastic polyp [8].

Previously, HP are commonly considered to be insignificant in terms of potential malignant conversion; however, some cases have been reported in which the hyperplastic foveal epithelium may develop to intestinal metaplasia, dysplasia, or carcinoma; this malignant transformation can occur spontaneously through the sequence hyperplasia-dysplasia-adenocarcinoma [9, 10].

On EGD, HP are seen as sessile or pedunculated elevated lesions. They can be unique or multiple, being located preferentially in the gastric antrum, but they may appear in any part of the stomach [2]. Most of these polyps are small (<1 cm); however in some cases they can be larger (>2-3 cm), taking a pedunculated or lobed appearance [7].

Magnifying endoscopy with FICE allows us to evaluate the surface structures and vascular structures of the mucosa. The margins of the cancer, in particular in case of differentiated carcinomas, are diagnosed on the basis of an irregular microsurface [11]. Histological examination showed a well-differentiated intramucosal tubular adenocarcinoma.

The incidence of malignant changes is relatively low, appearing mainly in those that measure more than 1 cm, being found focal intestinal metaplasia, dysplasia, and adenocarcinoma in 5-37%, 2-20%, and 2-6% of cases, respectively [12–14]. Carcinomas related to HP are usually well differentiated, although some cases of poorly differentiated carcinomas have been reported [9, 12].

It is suggested to resect HP if they are symptomatic, if they present atypical characteristics, or if they are greater than 1 cm, since they have a greater potential for malignant transformation [13]. When HPs measure less than 1 cm, biopsy and follow-up should be performed [12, 15]. It is also important to perform biopsies of the surrounding mucosa, to rule out the presence of gastritis associated with* H*.* pylori*, autoimmune gastritis and other pathologies [13].

In our review, there are no similar cases reported in our country and none in the literature that have endoscopic images with magnifying endoscopy with FICE. The present case demonstrates that some HPs may be associated with the presence of adenocarcinoma. Magnifying endoscopy with FICE may be helpful for this diagnosis. It is important that HP of more than 1 cm or symptomatic are resected. Endoscopic treatment of HP with adenocarcinoma is considered sufficient if the criteria for endoscopic and histological cure are confirmed [4, 15]. The follow-up should be done at 1 year and 3 years after polyp resection. In addition, a mapping of the gastric mucosa should be performed to determine the phenotype of gastritis associated with the presence of HP. Finally,* Helicobacter pylori* eradication is recommended if infection is found.
